# Complex benign horizontal canal positional vertigo: new perceptual management

**DOI:** 10.1016/j.bjorl.2022.05.003

**Published:** 2022-05-21

**Authors:** Narendra B. Suratwala, Jay N. Suratwala, Madhav V. Bapat

**Affiliations:** a VAV Care Clinic & Sarvoday Hospital, Surat, India; b Pune University, Department of Pathology, India

**Keywords:** Benign paroxysmal positional vertigo, Horizontal semicircular canal, Particle repositioning maneuver, Geotropic nystagmus, Apogeotropic nystagmus

## Abstract

**Objective:**

Horizontal semicircular canal site pathology of benign paroxysmal positional vertigo demonstrating three types of nystagmi on positional test were studied. We have attempted to design a protocol for its diagnosis and treatment.

**Methods:**

320 patients of HSC-BPPV were subjected to two types of positional tests. Of these, patients with bilateral steady apogeotropic nysatgmus were treated with VAV modification of Semont’s maneuver. Patients with unsteady or changing apo/geotropic signs were converted into steady geotropic ones by repetitive positional tests; followed by barbecue maneuver with forced prolong positioning.

**Results:**

Overall 88% of patients had a total recovery. 92% of patients with geotropic nystagmus showed no symptoms after second maneuveral sitting. 85% of patients with apogeotropic nystagmus recovered fully after third maneuveral sitting.

**Conclusions:**

Correct identification of subtypes of HSC-BPPV is based on provoked nystagmus by positional tests. After locating the site and side on the basis of nystagmic pattern, physician can apply the appropriate PRM.

**Level of evidence:**

II a.

## Highlights

• The maiden HPE report of HSC pathology improves the understanding of etiology of BPPV.
• The quick head rotational test using gravity complements Dix-Hallpike test and is more effective in locating cupular and anterior arm pathology of HSC.
• Modified II stage of Semont’s maneuvere using utmost gravity force clears the cupolar deposits more efficiently than concurrent therapies.
• The proposed protocol in managing HSC BPPV of complex presentation is found to be extremely useful in clinical practice in a busy vertigo clinic.

## Introduction

Benign Positional Paroxysmal Vvertigo (BPPV) is a common cause of dizziness, particularly in people older than 60 years. Its incidence is estimated to be 6000/million of population in those whose illness persists longer than 30 days, although this is probably a gross estimate. BPPV is easily identifiable with bed side clinical test (Dix-Hallpike). All three semicircular canals (anterior/superior, posterior, horizontal/lateral) may be the source of BPPV, although the literature quotes the posterior semicircular canal as the most common source. Various theories exist regarding the pathogenesis and localization of BPPV. Calcium carbonate embedded in proteinaceous matrix on surface of utricle,^1^ along with otoconia act as a building block. By virtue of head trauma, infection, and inflammation or idiopathicity, otoconia can dislodge and move to various labyrinthine channels within endolymphatic space. The pathological movement of otoconia induces disproportionate sensation of movement to that of actual head movement.

Variants of BPPV^2^ has been described in medical literature, although their existence is controversial, much debate has occurred on the etiological subject of as: ampulolithiasis (short arm BPPV or canalolithiasis) vs. Cupulolithisis (otoconia attached to cupula)^2^, ^3^ and utriculolithiasis.

In recent time a specific attention is made to detect BPPV related to Horizontal Semicircular Canal (HSC). The HSC variant of BPPV (HSC-BPPV) is usually induced by Hallpike-Dix test and or by head rotation in plane of HSC with head hanging at 30°‒45°.^2^ It can also be evoked by flexion or extension of head or from supine to up-right position.

Depending on the appearance of induced nystagmus; following variants of HSC-BPPV can be identified and classified into three pathological subgroups viz: 1) Most common type is bilateral geotropic nystagmus where Free Floating Particles (FFP) detached from utricular macula travelling in post arm of HSC; 2) Second common type, which shows bilateral unstable nystagmus (Changing from apogeotropic to geotropic after few Particle Re-Positioning Maneuvers (PRM). Pathophysiology may be related to the shift of FFP from anterior arm of HSC to posterior arm of HSC; 3) Third type, which can be evoked in form of bilateral stable or fixed apogeotropic nystagmus, can be interpreted as a cupulolithiasis.^4^

Of the above three types described, the third type shows most aggressive longer lasting symptoms often accompanied with neuro-vegetative phenomena (Nausea, Vomiting, involunatry passage of urine or stools with perspiration).^2^ Compared to canalithiatic pathology (short lasting nystagmus of less than 20 s) the nystagmus evoked in cases of cupulolithiasis is lasting for more than two minutes.

In spite of the listed signs and symptoms above, at present we lack in producing the model which can navigate to produce standard protocol for treatment of HSC-BPPV unlike that of PSC-BPPV: as Epley’s maneuver is not effective to cure HSC-BPPV.

First undocumented attempt to remove FFP from HSC was done by Vannucci et al.^3^ by way of head shaking for 20 times in a horizontal plane in supine position with head lifting by 30°. In 1994, Lempert^5^ proposed “Barbecue rotation” based on 180° rotation from supine to prone position towards the affected ear. This was later modified by him in to 360° roll over. In the same year, Vannucchi achieved good results with Forced Prolonged Positioning (FPP). Later Epley^6^ in 1995 propose Barbecue maneuver in 90° steps (unreported-results). After reviewing the literature for each type of PRMs’ outcome related to HSC-BPPV it can be said that symptom free percentage of patients are up to 70% which is valued lower than that of cases relieved by Epley’s PRM for PSC-BPPV.^7^, ^8^, ^9^

As the abundant literary articles have proved by describing various PRMs to be more effective and quicker in curing the BPPV pathology when compared with natural recovery^10^, ^11^, ^12^ thence; the presented prospective and randomized study is conducted in a single medical centre. The aim of our study is to assess all three types of evoked nystagmus (vide supra) with various positional tests (vide infra) in cases of suspected HSC-BPPV, co-relating these signs with the elicited history and identifying the probable site of pathology in HSC and performing different PRMs (with or without modification) to come out with better outcomes.

### Case report

**Figure 1:**
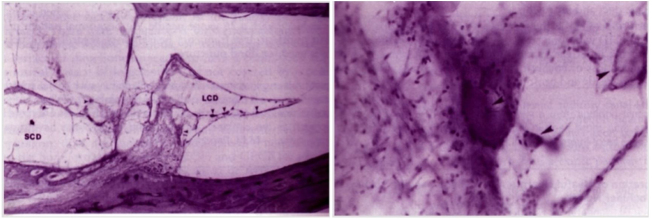
(A) HPE of Cupulolithiasis of Left temporal bone with haematoxyline-eosin staining reveals fixed and free otolith particles in HSC (LSC) under 40× magnification. (B) under 145× magnification reveals fixed particle clinged onto cupula.

A healthy male 42 yrs. old approached us in February 2002 with history of recurrent episodic vertigo. The vertigo was often triggered with change in bed position from supine to left lateral and lasting for 2–3 min. The vertigo was lessened with lying on supine. He preferred to lie down on right lateral position during sleep. His systemic and ear, nose, throat, systemic and neurological examination was unremarkable. The bedside Hallpike positional test done repeatedly showed changing nystagmus (apogeotropic as well geotropic) pattern in horizontal axis. Patient was put on intermittent short therapy of vestibular suppressants and vestibular adaptive exercises for 3 months which failed to improve the vertigo. In May 2002 he met with an fatal road accident and died of severe chest compressive injury. His temporal bone autopsy specimen was available for HPE study which revealed the adherent “debris” to HSC cupula as well as few free particles in membranous canal portion (Fig. 1).

## Methods

This institutional study has been granted an exemption by ethical committee by document number IEC/SH/08/1995 which is in compliance with Helsinki declaration (1964) as well with (ICMR guidelines 1990). From May 1995 till October 2019, we examined 320 consecutive patients were diagnosed to be of purely HSC-BPPV origin on the basis of positional tests in a central city vertigo clinic. Duration of symptoms ranged from 15 to 770 days (average duration 65 days). All patients described brief episodic intense vertigo while changing the positions in bed as lateral to supine and vis-a-vis with the duration of intense phasic vertigo lasting between few seconds to 2 min. Pure Horizontal positional nystagmus was seen during Dix-Hallpike test in majority of the cases and in modified head rotational positional test (VAV positional test (named so, as it is introduced at Voice and Vertigo Clinic), vide infra) (Table 1, Fig. 4). This modification is based on hypothesis of using sudden gravitational force (Jerk to HSC Cupula) resulting in to dislodgement of ‘fixed or adherent particles’ (debris) from cupula and makes it to travel along the course of HSC towards utricle via short arm. Neurological assessment with occasionally supported by Brain imaging is done to confirm the absence of central nervous pathology which could explain the positional nystagmus; for better understanding and recording purposes we use computer based Videonystagmography (VNG).

**Table 1:** Showing initial and/final comparative outcomes of two different positional tests in HSC BPPV (n = 320).

Inference	Dix-Hallpike test	VAV positional test	Accord numbers	Final Site location after pooled test results
Canalithiasis	193	165	155	190
Cupololithiasis	117	148	105	130
No nystagmus	10	07	‒	0

**Figure 2:**
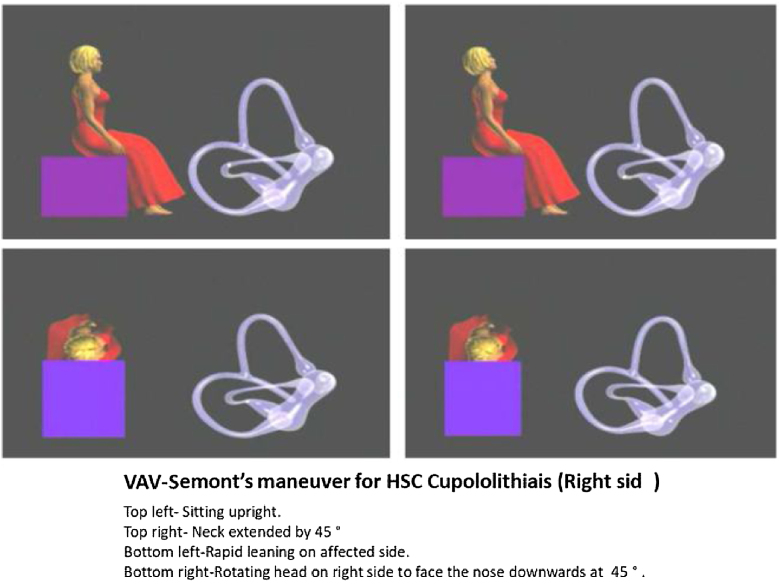
Describing the steps of VAV-Semont’s PRM for Cupulolithiasis.

Before performing specific physical therapy (PRM),^13^ we re-confirmed the side and site of lesion based on nystagmus characters and matching it with clinical history and noting down initial and final observations on nystagmus (Table 1) At a time we did try to convert apogeotropic nystagmus into geotropic by performing VAV-Semont maneuver (Fig. 2) (modified at VAV Care Clinic and so named after) or by repeated VAV head rotational positional test. All the induced nystagmus findings in the subjects were recorded as classified into 1) Steady apogeotropic (Cupulolithiasis); 2) Steady geotropic (Canalolithiasis); 3) Transformable from apogeotropic to steady or persistent geotropic.

PRM was performed as:

1) For HSC cupulolithiasis VAV-Semont’s (modified II stage of Semont’s) maneuver was done. Patient sits upright, neck then extended to 45° for 10 s and then made to quickly lie down on the affected side followed by rotating the head by 45 ° to return to the starting downwards facing the earth and maintaining this position for 3–5 min and then bringing him back to upright position. Usually, patient reports relief from head heaviness (Fig. 2). If not, the PRM was repeated with head shakes (20 times on each side with neck extended from supine position at 45°.
2) For HSC canaloithiasis, Barbecue maneuver is performed at home or in wards with his headfirst from affected side to normal side followed by trunk rotating in each step by 90° in a sequence from healthy side to affected side (lateral, prone, opposite-lateral, supine, lateral at the starting position). Each step is followed by 2 min pause, after attaining final lateral position on healthy side patient is asked to maintain FFP for 10 h.

All the maneuvered subjects were asked to report the details of recovery on our media network after 48 h and at 10 days, 6-months and 2-years: those non-beneficiaries were asked to attend repeat physical therapy.

Few non-responders to PRMs were put on intermittent vestibular suppressant and Vestibular Adaptation Exercises (VAE).^1^

## Results

Of 320 healthy individuals with female: male ratio of 3:2 (192 females vs. 128 males) reported short lasting episodic rotatory vertigo. Of these 63 subjects had neuro-vegetative symptoms. Vertigo induced from supine to lateral was present in 210 subjects and from sitting to lying down (vis-a-vis) in 55 subjects in their history, further 46 subjects reported the onset of vertigo with either change in posture; majority of these reported to be at ease, during episodic vertigo with eyes closed. There was some predilection of Cupulolithiasis occurring more in females compared to males with neuro-vegetative symptoms.

Dix-Hallpike and VAV tests were applied randomly in 320 suspected HSC-BPPV patients; shows 117 cases of cupulolithiasis and 193 cases of canalolithiasis were inducible by Dix-Hallpike test, while 148 cases of cupulolithiasis and 165 cases of canalithiasis were inducible by VAV test (Table 1). When combined tests were performed in this group of patients with positive nystagmus signs; finally, 190 cases were of cupulolithiasis and 130 cases were of canalolithiasis were labeled on the basis of final analysis of both positional test (Table 1).

Initial observation was with reports of 130 cases with geotropic and 190 cases with apogeotropic forms of nystagmus after combination of both type of positional test. Final observation listed before applying PRM was as; canalolithiasis with geotropic nystagmus presentation were 190 and with apogeotropic nystagmus were 130 (Table 1); hence 60 cases were labeled as “direction changing nystagmus” from apogeotropic to geotropic ones. There was no occurrence of shifting of geotropic to apogeotropic nystagmus.

The side of lesion was labeled after closely co-relating the history with findings of positional tests (triggering side, amplitude, and slow phase velocity on VNG).

PRM was applied as: VAV-Semont’s maneuver in all 130 of cupulolithiatic cases (with assisted head shakes only in 20 cases) and Barbecue maneuver with FFP for 10 h in 190 Canalolithiatic cases. Complete resolution was noted in 77% (105/130) in cases of cupulolithiasis after 1st PRM and in 85% (114/130) after 3rd PRM. Canalolithiatic group showed complete resolution in 87% (165/190) after 1st PRM with FPP and 90% 174/196) after 2nd PRM with FPP.

## Discussion

Benign paroxysmal positional vertigo represents 15%–22% of all otogenic dizziness.^1^, ^14^ The senior author himself has noticed it as 10%–12% of all other causes of vertigo. Again, in recent time, our clinic is able to see the rise in incidence of HSC-BPPV due to better understanding of labyrinthine pathology and improved diagnostic ability with the use of various positional tests (Table 1).

The challenges in managing HSC-BPPV are: 1) Detection of affected side; 2) Detection of sites; short arm of HSC vs. long arm of HSC; 3) Deciding the sequential steps to elicit the nystagmus; 4) Choosing the correct PRMs to yield optimum results.

**Figure 3:**
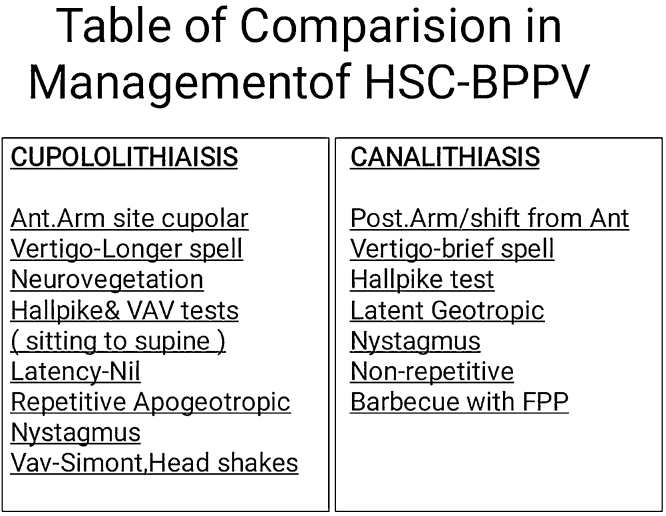
Describing differences in clinical signs and treatments of cupulolithiasis.

These are met successfully as:

1) Side detection can be decided on basis of correct history taking and type of nystagmus which is bilateral horizontal beating with apo/geotropic with non/fatiguing (Fig. 3). At a time, evaluating the difference in subjective symptoms and nystagmus amplitude on vestibuloculogram^2^ is useful guide to predict the affected side. In patients with geotropic nystagmus induced by rolling the head to affected side provokes ampullopetal deviation of cupula; when the patient rolls the head to healthy side FFP displacement makes ampullofugal cupular displacement (Ewald’s 2nd law) to result in to less intense geotropic inhibitory nystagmus.^2^ Even the presence of short lasting nystagmus from sitting to supine position in repetition provides a hint of eventual inhibitory nystagmus.

**Figure 4:**
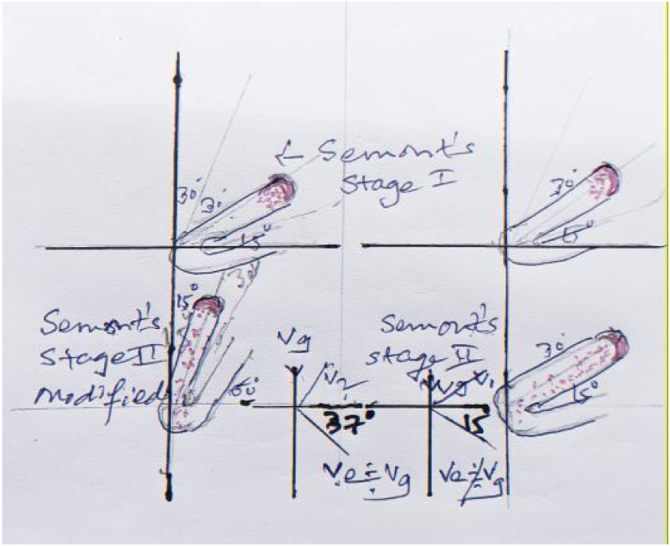
Physicist’s and artist’s impression describing: 1) Right side Semont’s maneuver stage II results in to impingment of FFP at bend between anterior and posterior arm of HSC due to obtuse angulation at ampulla resulting in to effective vector (Ve- with acute angulation to horizontal plane) which fails to propel the debris to posterior arm, as opposed to 2) Left side diagram with stage II of Semont’s maneuver modified resulting in to effective vector (Ve) near to Gravity force (Vg) with less acute angulation to the horizontal plane enabling better clearing of the debris in HSC- Cupulolithiasis.
2) Site in HSC can be decided again on evoked apo/geotropic nystagmus and consistent/non consistent (refer hereafter as stable/unstable) (i.e., non/direction changing type). Stable apogeotropic feature indicates copular lesion while unstable apogeotropic^15^ indicating FFP in longer arm and stable geotropic feature suggest FFP in short arm.
3) We found following steps to be effective to evoke the nystagmus optimally. a) Cessation of the vestibular suppressants for 48 h. b) Correct history pertaining to the details of episodic vertigo (Duration of intense phase, associated symptoms, triggering and relieving factors. c) Performing Dix-Hallpike and VAV positional tests with 2–3 times repetition and recording with VNG. In case of un-stable apogeotropic nystagmus we tried to convert it into stable geotropic one (FFP travelling from longer to short arm-with ampullofugal endolymphatic flow); by repeating VAV test or occasionally doing VAV-Semont's maneuver. This was possible due to bringing gravitational factor to produce effective elimination of FFP along the effective vector created nearing to gravity vector (Vg = Ve, Fig. 4) in to action which also explains the rationale behind performing VAV positional test (Figs. 2‒4).

Yun-Hoon Choung^16^ in 2006 compared the efficiency between the classic method and BLT in 26 patients with HSC-BPPV. The classic method is based on Ewald’s second law comparing the intensity of nystagmus or symptoms in the head roll test. BLT is based on the direction of both “bowing nystagmus” and “leaning nystagmus” at the head’s bowing and leaning state in a sitting position. The affected ear is the same direction of bowing nystagmus in canalolithiasis and the same direction of leaning nystagmus in cupulolithiasis. Further he reports, In 26 patents (15 canalolithiasis, 11 cupulolithiasis), 3 (11.5%) patients did not show a prominent affected ear in the classic method, and 7 (26.9%) patients showed the different affected ear between the two methods; while in our study Dix-Hallpike test as well VAV positional test showed the insensitivity for induced nystagmus is 3% and 2% respectively; with no discord for deciding the affected side (Table 1).

**Figure 5:**
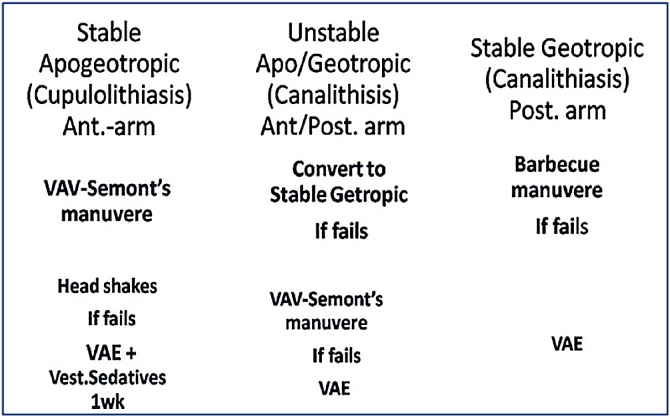
Displaying the proposed Protocol in management of HSC-BPPV.

4) Once these steps are done PRM is selected on basis of protocol (Fig. 5).

Patients with stable apogeotropic (Cupulolithiasis) are treated with VAV-Semont’s PRM. If it fails, then head shakes is implemented followed by repeating of VAV-Semont’s PRM. Failing this, the patient is put on to short duration (2–4 days) of vestibular suppressants and VAE.

Unstable apo/geotropic nystagmus is tried to be converted preferably in to stable geotropic following which barbecue PRM^6^, ^9^, ^17^ is performed. In case the nystagmus remains as stable (less common to happen) then we proceeded with VAV-Semont’s PRM. Failing this, the subjects were advised to undergo VAE with intermittent short time use of vestibular suppressants.

The stable geotropic nystagmus showing subjects were treated with barbecue PRM and FPP for 10 h.^8^ Failing this, patients were put on to VAE without vestibular suppressants as these subjects rarely only report severe vertigo.

Literature has expressed possible fear of FFP migration to other canals or sites. We haven’t come across any such incidental finding in 320 individuals.

The strategic treatment reported in this series of HSC-BPPV has resulted complete resolution of vertigo over 88% as against 42% in control group^3^, ^14^ from other studies at 12 weeks. Ramos B.F.^18^ reported total resolution of vertigo and positional nystagmus in all seven subjects of geotropic induced nystagmus in a single sitting with Zuma modified maneuver. Results of this series (success rate in 92% canalithiasis and 85% in cupolithiasis) are comparable to study report of A.P.Casani^2^, ^6^ (success rate 90% in canalolithiasis and 65% in cupololithiasis) and better over the study reports of Apiani^8^ and Nuti^3^ who used FPP technique and nearly to Epley’s^19^ method for PSC-BPPV.

## Conclusion

In the last century, treatment with diagnosing ability looked bizarre. Diagnosing abilities in suspected BPPV has been greatly improved upon in last 2 decades. The Senior author witnesses the shifting paradigm in the management of BPPV in recent time; especially after considering maiden evidence of cupulolithiasis on HPE in a case. Hence, on the basis of fixed cupular particles or FFP one is able to locate the side and site of culprit attributed to a troublesome recurring peripheral vertigo. The key factor in the management of HSC-BPPV are well studied nystagmus pattern in co-relation to correctly expressed history and choosing the correct maneuver pertaining to final observation made of combined positional test (Fig. 5). The protocol suggested above has been a useful guide for us to yield the total recovery in 88% of patients.

## Conflicts of interest

The authors declare no conflicts of interest.
